# CLEVER-1 targeting antibody, bexmarilimab, supports HLA-DR expression and alters ex vivo responsiveness to azacitidine and venetoclax in myeloid malignancies

**DOI:** 10.1038/s41598-025-01675-y

**Published:** 2025-05-14

**Authors:** Sofia Aakko, Arno Ylitalo, Heikki Kuusanmäki, Jenna H. Rannikko, Mari Björkman, Jami Mandelin, Caroline A. Heckman, Mika Kontro, Maija Hollmén

**Affiliations:** 1 Faron Pharmaceuticals Ltd, Turku, Finland; 2 Institute for Molecular Medicine Finland, HiLIFE, University of Helsinki, Helsinki, Finland; 3 MediCity Research Laboratory and InFLAMES Flagship, University of Turku, Turku, Finland; 4 Biotech Research & Innovation Centre (BRIC) and Novo Nordisk Foundation Center For Stem Cell Biology (DanStem), University of Copenhagen, Copenhagen, Denmark; 5 Foundation for the Finnish Cancer Institute, Helsinki, Finland; 6 Department of Hematology, Helsinki University Hospital Comprehensive Cancer Center, Helsinki, Finland

**Keywords:** Acute myeloid leukemia, Myelodysplastic syndrome, Bexmarilimab, CLEVER-1, Stabilin-1, Immunotherapy, Cancer, Haematological cancer, Tumour immunology

## Abstract

Treatment of myelodysplastic syndrome (MDS) and acute myeloid leukemia (AML) requires new therapy options, especially for patients uneligible for intense chemotherapy or with relapsed or refractory disease. CLEVER-1 is a myeloid checkpoint protein, which can be targeted with a therapeutic function blocking antibody, bexmarilimab. Bexmarilimab has shown clinical efficacy in different solid tumors. Here, we show preclinical data demonstrating expression of CLEVER-1 on immature malignant myeloid cells and their derivates in MDS and AML bone marrow samples and AML cell lines. Highest CLEVER-1 levels were observed in AML with monocytic differentiation. Ex vivo treatment of AML/MDS bone marrow samples with bexmarilimab led to an increase in antigen-presenting human leukocyte antigen DR isotype (HLA-DR) molecule expression. Combination of bexmarilimab with current standard-of-care (SoC) drugs, azacitidine and venetoclax, showed potential for HLA-DR induction and enhanced killing of leukemic cells, respectively. Our non-clinical findings support the feasibility of CLEVER-1 inhibition in AML/MDS to induce antigen presentating molecule expression and potentially, an anti-leukemic effect together with SoC. Therapeutic targeting of CLEVER-1 with bexmarilimab is currently undergoing clinical investigation in the BEXMAB trial (NCT05428969).

**Supplementary Information:**

The online version contains supplementary material available at 10.1038/s41598-025-01675-y.

## Introduction

Hematological malignancies of the myeloid lineage, such as myelodysplastic syndrome (MDS) and acute myeloid leukemia (AML) remain difficult to treat, especially after disease relapse. Immunotherapies, such as programmed death(-ligand) 1 (PD-(L)1) inhibitors and chimeric antigen receptor T cells have not shown efficacy against myeloid malignancies in contrast to many other neoplasms. Complex immune environment in the bone marrow (BM), immune evasion, low mutational burden and lack of universal leukemia-specific antigen(s), are potential reasons for the poor outcome of immunotherapies in myeloid diseases^1,2^. Especially, immunosuppressive macrophages in the leukemic BM drive leukemogenesis and therapy resistance in myeloid malignancies. Although the AML BM macrophage population shows substantial heterogeneity, enrichment of M2-like macrophages associates with dismal prognosis and disease relapse^3–5^. M2-like macrophages have been shown to support leukemia cell proliferation via promoting selection of stem-cell like leukemic cells and modulating blast cell metabolism^3^. On the other hand, leukemic blasts can polarize BM macrophages to M2-like phenotype to promote a leukemia permissive BM environment^6^.

Reprogramming macrophages as a therapeutic strategy for hematological malignancies has attained increasing interest. A feasible target in this setting is the scavenger receptor CLEVER-1 (also known as FEEL-1 and Stabilin-1), expressed on immunosuppressive macrophages, circulating monocytes, as well as sinusoidal and vascular endothelium^7^. In vitro blocking of CLEVER-1 on the myeloid immune cells inhibits the binding and uptake of acetylated-low density lipoprotein (Ac-LDL)^8^. This blocks the activation of nuclear lipid pathways (liver X receptor-retinoid X receptor, LXR/RXR and peroxisome proliferator-activated receptor, PPAR) and supports pro-inflammatory nuclear factor kappa B (NF-κB) signaling and release of tumor necrosis factor alpha (TNF-α)^8,9^. Additionally, inhibition of CLEVER-1 impairs the endolysosomal acidification and antigen degradation, enhancing antigen cross-presentation. Hence, CLEVER-1 deficient mice show increased frequency of tumor associated macrophages with high major histocompatibility complex (MHC) II protein expression^9,10^ These changes align with macrophage reprogramming and innate immunity activation, leading to release of other proinflammatory cytokines (e.g., interferon gamma (IFNγ) and activation of adaptive immune system (T cells)^11,12^. This immune activation results in anti-tumor activity in mouse models^10^ and clinical benefit in patients with advanced solid tumors^12^ treated with a therapeutic humanized anti-CLEVER-1 IgG4 antibody (FP-1305, bexmarilimab).

Since macrophages and malignant blasts originate from the same leukemic myeloid progenitor clone, myeloid malignancies are the only cancer type where CLEVER-1 may be also expressed by the malignant cells. Indeed, high expression of *STAB1 messenger RNA (mRNA)*, encoding CLEVER-1 protein, has been suggested to be a marker for poor prognosis in cytogenetically normal AML^13^. Knockdown of *STAB1* inhibits AML cell line proliferation and growth in xenograft mice, possible via altering NF-κB pathway activity and has been suggested to increase AML cells sensitivity to venetoclax^13,14^. In AML and MDS, inhibition of CLEVER-1 may have direct anti-leukemic activity or lead to modulation of myeloid cells and the bone marrow immune environment to improve the efficacy of other therapies.

Here, we characterize the role and therapeutic potential of CLEVER-1 in AML and MDS, beyond its’ macrophage checkpoint function. We show that AML cell lines and malignant myeloid bone marrow cells of AML and very high risk MDS patients express CLEVER-1. By inhibiting CLEVER-1 in cell lines and patient cells ex vivo with bexmarilimab treatment as single-agent and in combination with azacitidine and venetoclax, we demonstrate changes in myeloid cell antigen presentating molecule expression and leukemia cell sensitivity to SoC drugs.

## Methods

### Cell line viability measurements

At FIMM, AML cell lines were cultured in RPMI + 10% FBS (HNT34, GDM1, HEL, MOLM13), RPMI + 10% FBS + 2ng/mL GM-CSF (TF1), RPMI + 20% FBS (CMK, Kasumi1, SKM1), RPMI + 20% FBS + 10ng/mL GM-CSF (F36P), IMDM + 20% FBS (KG1) or AlphaMEM + 20% FBS (OCI-AML2). Cell amount optimized for cell proliferation measurement at 72 h post-treatment was plated (1250–5000 cells/well; 384-well plate). Cell Titer-Glo (Promega, WI, US) was used per kit instructions to study cell proliferation after 72 h treatment with bexmarilimab (Bex) or IgG4 control (0.1-60ug/ml) or with Bex/IgG4 in combination with azacitidine (1 μm) and/or venetoclax (50nM).

At MediCity, KG-1 cell line was cultured for 7 days in StemSpan SFEM II (STEMCELL Technologies, Vancouver, CA) medium with a CD34 + expansion supplement and treated with bexmarilimab (50ug/mL) together with dimethylsulfoxide (DMSO; matching v/v %) control or azaciditine (1 μm) and/or venetoclax (50nM). Human IgG4 was used as a non-targeting control for bexmarilimab treatment. Azacitidine /DMSO was added at 48-hour intervals. Cell viability was measured after a 7-day incubation using AlamarBlue (ThermoFisher Scientific, MA, US) assay.

### Bone marrow samples

All AML (*n* = 34) and MDS (*n* = 4) patient samples were provided by the Finnish Hematology Registry and Biobank and used in accordance with the Declaration of Helsinki. Mononuclear cells had been extracted and frozen per the biobank standard practice. The samples were analysed at two independent laboratories; 14 AML samples at MediCity (Medicity cohort 1, *n* = 8 samples and cohort 2, *n* = 6 samples) and 20 AML and 4 MDS samples at FIMM (FIMM cohort). Sample characteristics are listed in Suppl. Table 1. Following sample thawing, slightly divergent procedures were followed for culture conditions and drug treatments at MediCity and FIMM. For MediCity cohorts 1 and 2, 40 000 cells/well were seeded on a 96-well plate in IMDM medium supplemented with 1% FCS and treated with 50ug/ml Bex and irrelevant human IgG4 as control. In FIMM cohort, samples were treated with DENARASE (c-Lecta, Leipzig, DE), let recover for 4 h and 50 000 or 100 000 cells/well were plated on pre-drugged 96-well plates, depending on total viable cell yield after thawing. For FIMM cohort, 12.5% Hs-5 derived conditioned medium with RPMI/10% FBS/2mM L-glutamine base was used to optimize primary cell viability and *ex vivo* drug sensitivity testing^15,16^. RBC lysis was performed before plating upon suspected red blood cell contamination. FIMM samples were treated with 50ug/ml Bex and control IgG4 in combination with DMSO (matching v/v%), azacitidine (300 nM or 1 μm), venetoclax (50 nM) or 300nM azacitidine/ 50nM venetoclax. Bex and IgG4 were added on the plates briefly prior to cell plating.

### Flow cytometry

For the patient samples, flow cytometry profiling was performed both at 0 h from untreated cells and after 48 h drug treatment in both cohorts. Antibodies used for cell staining are listed in Suppl. Table 2. A cell viability dye was used in all panels to exclude dead cells from the analysis. Data was acquired with BD LSR Fortessa (BD, NJ, US) or iQue Screener Plus (Intellicyt, NM, US) and analysed with FlowJo v10.8.0 or Forecyt at MediCity and FIMM, respectively. Similar gating strategies were followed at both MediCity and FIMM. Blasts, monocyte-like and lymphocyte populations were gated based on CD45-SSC and subpopulations characterized further based on CD34 (blasts), CD14/CD16 (monocytes) and in MediCity, also CD11b (monocytes) positivity (from CD45 + cells) (Fig. S1). CLEVER-1 and HLA-DR expression was analysed only from populations that were > 5% of the primary population (CD45 + or live) and/or had > 100 events in IgG4 control well, from FIMM and MediCity cohorts, respctively. Relative viability for the gated cell populations was determined by normalizing the absolute number of viable cells present after treatment to the number of cells present in the wells treated with IgG4 control. Determination of intracellular CLEVER-1 expression in the MediCity cohort 2, was performed following the same methodology as for cell surface CLEVER-1 staining, except a permeabilization step was added after staining the other surface markers.

### Multiplex

Cytokines from conditioned media were measured using Bio-Plex Pro Human Cytokine 27-plex assay (Bio-Rad, cat. M500KCAF0Y) and Bio-Plex 200 System (Biorad) according to the manufacturer’s instructions.

### NF-κB reporter assay

THP1-Lucia™ NF-κB (InvivoGen, CA, US) reporter cell line was used to study the contribution of bexmarilimab-mediated IFNγ induced signalling pathways. 10 000 cells/well were seeded in 96-well plates in RPMI 1640, 2 mM L-glutamine, 25 mM HEPES, 10% (v/v) heat-inactivated fetal bovine serum (FBS; 30 min at 56 °C), 100 U/ml penicillin, 100 µg/ml streptomycin, 100 µg/ml Normocin™. Bexmarilimab or IgG4 were added at 10ug/mL and incubated on cells for 18–24 h. Cells were pre-incubated with recombinant CLEVER-1 fragment (H1; Icosagen, Õssu, EE) at 20ug/mL when testing for specificity of bexmarilimab-mediated activation of NF-κB promoter. For the NF-κB promoter activity readout, 10 µL of cell culture supernatants were transferred to a 96-well white opaque plate, to which 50 µL of QUANTI-Luc™ Reagent (InvivoGen) was added and luminescence was measured using Tecan Infinite plate reader with end-point measurement (4 s start time and 0.1 s reading time) immediately.

### Statistical analyses

#### Flow cytometry and cell line viability data

For correlation analyses, either Pearson or Spearman correlation coefficient was calculated, depending on the normality of distribution. Statistical significance between control and bexmarilimab treated groups was tested with paired t-test or Wilcoxon matched pairs signed rank test. One-way (repeated measures) ANOVA + Dunnet’s multiple comparison test or Kruskal-Wallis test + Dunn’s post hoc test were used to test the difference between more than two groups, depending on the normality of distribution in the data.

#### RNA-seq data

*STAB1 mRNA* expression was studied from BEAT-AML 2.0 and TCGA-LAML datasets. Analyses were performed with R (v.4.0.4). For BEAT-AML dataset analyses, conditional quantile normalized (log_2_RPKM) RNA-seq data and sample metadata were downloaded from https://biodev.github.io/BeatAML2/. Additional sample metadata was derived from Bottomly et al. (2022 Cancer Cell, Suppl. Table S1). RNA-sequenced bone marrow aspirate samples from patients with diagnosis ACUTE MYELOID LEUKAEMIA (AML) AND RELATED PRECURSOR NEOPLASMS at specimen acquisition and with RNAseq sample annotated as “rna_include_in_analysis” in the downloaded metadata were used in the analyses. For TCGA-LAML dataset analyses, RNA-seq data and patient clinical data were downloaded from the Genomic Data Commons (GDC) Data Portal (https://portal.gdc.cancer.gov/projects/TCGA-LAML, https://gdc.cancer.gov/about-data/publications/laml_2012). Obtained FPKM-UQ-normalized counts were log_2_-transformed and samples filtered to exclude normal tissue samples. To match the two datasets with our flow cytometry analyses, only FAB categories M0-M2 and M4-M5 were included (BEAT-AML: *n* = 192; TCGA-AML: *n* = 132). For comparing *STAB1* mRNA expression between two groups, Wilcoxon rank-sum test was used.

## Results

### Myeloid leukemia cells express CLEVER-1

**Fig. 1:**
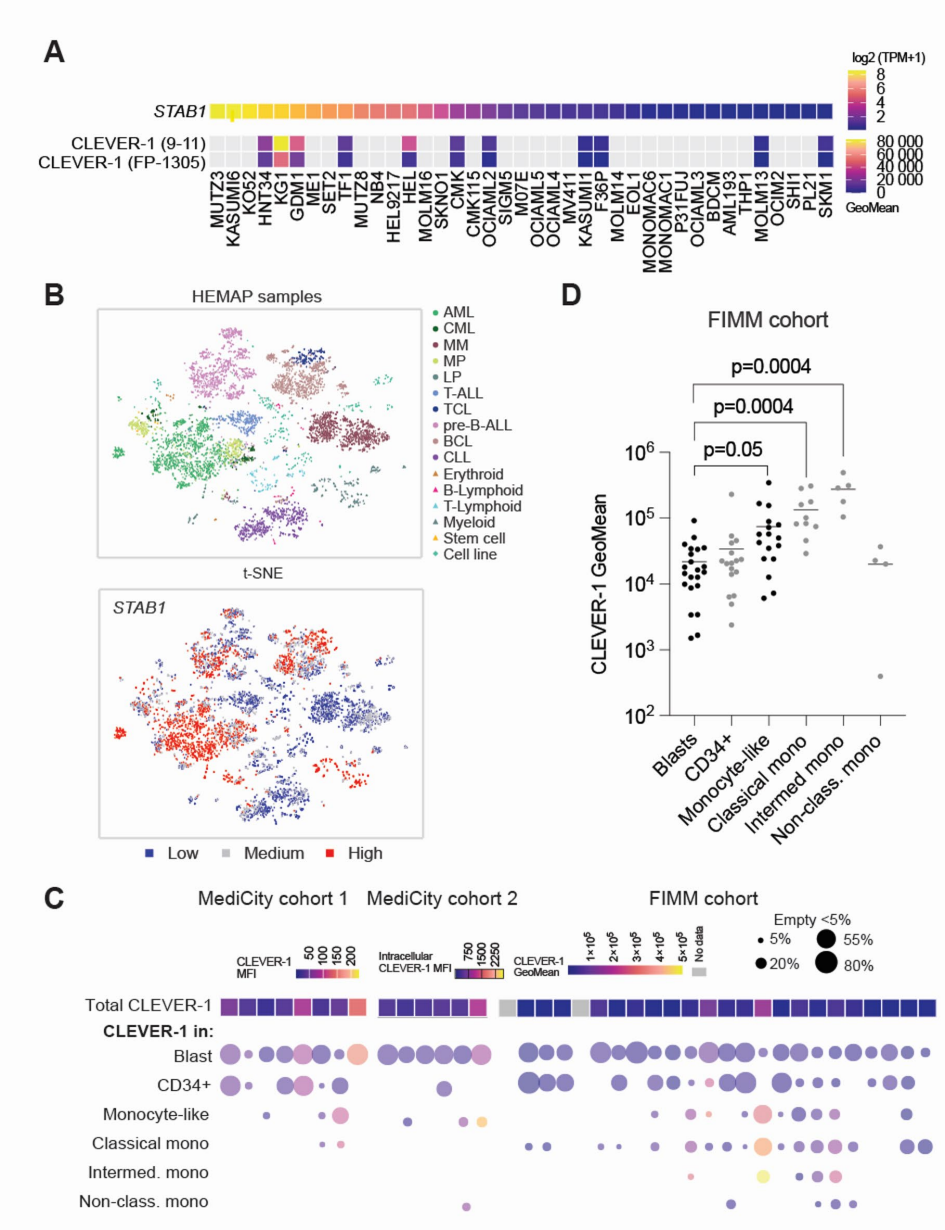
CLEVER-1 is expressed on malignant myeloid cells. (**A**) *STAB1* RNA expression in 39 AML cell lines, presented in a heatmap as log2 values, derived from DepMap and cell surface CLEVER-1 protein expression in 11 AML cell lines, detected with two antibodies that recognize different epitopes of CLEVER-1 and presented as GeoMean in a heatmap. (**B**) *STAB1* RNA expression in primary samples representing different hematological (pre)malignancies and cell types, derived from the Hemap database. AML = acute myeloid leukemia, CML = chronic myeloid leukemia, MM = multiple myeloma, MP = myeloproliferative disease, LP = lymphoproliferative disease, T-ALL = T-cell acute lymphoblastic leukemia, TCL = T cell lymphoma, pre-B-ALL = pre-B-cell acute lymphoblastic leukemia, BCL = B cell lymphoma, CLL = chronic lymphocytic leukemia. (**C**) Total expression of surface or intracellular CLEVER-1 protein (in CD45 + leukocytes) and in different myeloid cell populations (bubble plot) of primary AML and MDS samples. Each column represents one sample. CLEVER-1 expression (Median Fluorescence Intensity, MFI or Geometric Mean Intensity, GeoMean) is indicated by the color of the rectangle (in CD45 + leukocytes) or circle (in different myeloid cell populations). Circle size reflects the size of the population as % from CD45 + cells in each sample. Lack of cell population specific CLEVER-1 expression is due to the cell population being too small for analysis (< 5% of CD45 + in control treated sample at 48 h). ‘Blasts’ (CD45loSSClo) and ‘monocyte-like’ (CD45hiSSClo/mid) cell populations are defined based on CD45-SSC, ‘CD34+’ (CD45 + CD34+) is a subpopulation of blasts and monocyte subpopulations are based on CD14/CD16 expression (Fig. S1). (**D**) Summary data for CLEVER-1 expression in different cell populations of FIMM cohort samples. Each dot is one sample, line shows cell population mean and grey-colored populations are sub-populations of blasts or monocyte-like cells. P-values are based on Kruskal-Wallis and Dunn’s test.

To study CLEVER-1 expression in malignant myeloid cells, we first checked the DepMap database, where a panel of 39 AML cell lines showed variable expression levels of *STAB1* mRNA. We measured CLEVER-1 protein expression in 11 of these cell lines and found it to be consistent with *STAB1* mRNA expression (Fig. 1A and Fig. S2A-B). No significant association between cell line FAB type and *STAB1* mRNA or CLEVER-1 protein expression was noted. Next we studied the Hemap^17^ database, including data from human samples of different hematological (pre-) malignancies. Among the different malignancies, the results indicated high expression of *STAB1* mRNA in nearly all AML samples and in a subset of the myeloproliferative disease samples (MDS), T- and B-cell lymphoma samples, as well as in pre-B-cell acute lymphoblastic leukemia (Fig. 1B). To confirm this finding, we measured CLEVER-1 protein expression in two independent laboratories with flow cytometry from 34 AML and 4 MDS primary bone marrow samples, divided into three cohorts described in Suppl. Table 1. Our results verified cell surface (MediCity 1 and FIMM) and intracellular (MediCity 2) CLEVER-1 expression on malignant myeloid cells (Fig. 1C). Leukemic blasts, including CD34 + blasts, expressed CLEVER-1 in all samples. The majority of CLEVER-1 resided in monocytes or monocyte-like cells, derived from the immature malignant myeloid cells. In myelomonocytic/monocytic AML the monocyte-like cell population may contain also more immature monoblast cells. From the different monocyte subpopulations, divided according to surface CD14/CD16 marker expression, the classical (CD14 + CD16-) and intermediate (CD14 + CD16+) monocytes showed highest CLEVER-1 expression based on the statistical analysis of the FIMM cohort data (Fig. 1D). Despite being a rare monocyte population, intermediate monocytes have important functions such as antigen presentation and T cell stimulation^18^.

### High CLEVER-1 expression in AML with monocytic differentiation

**Fig. 2:**
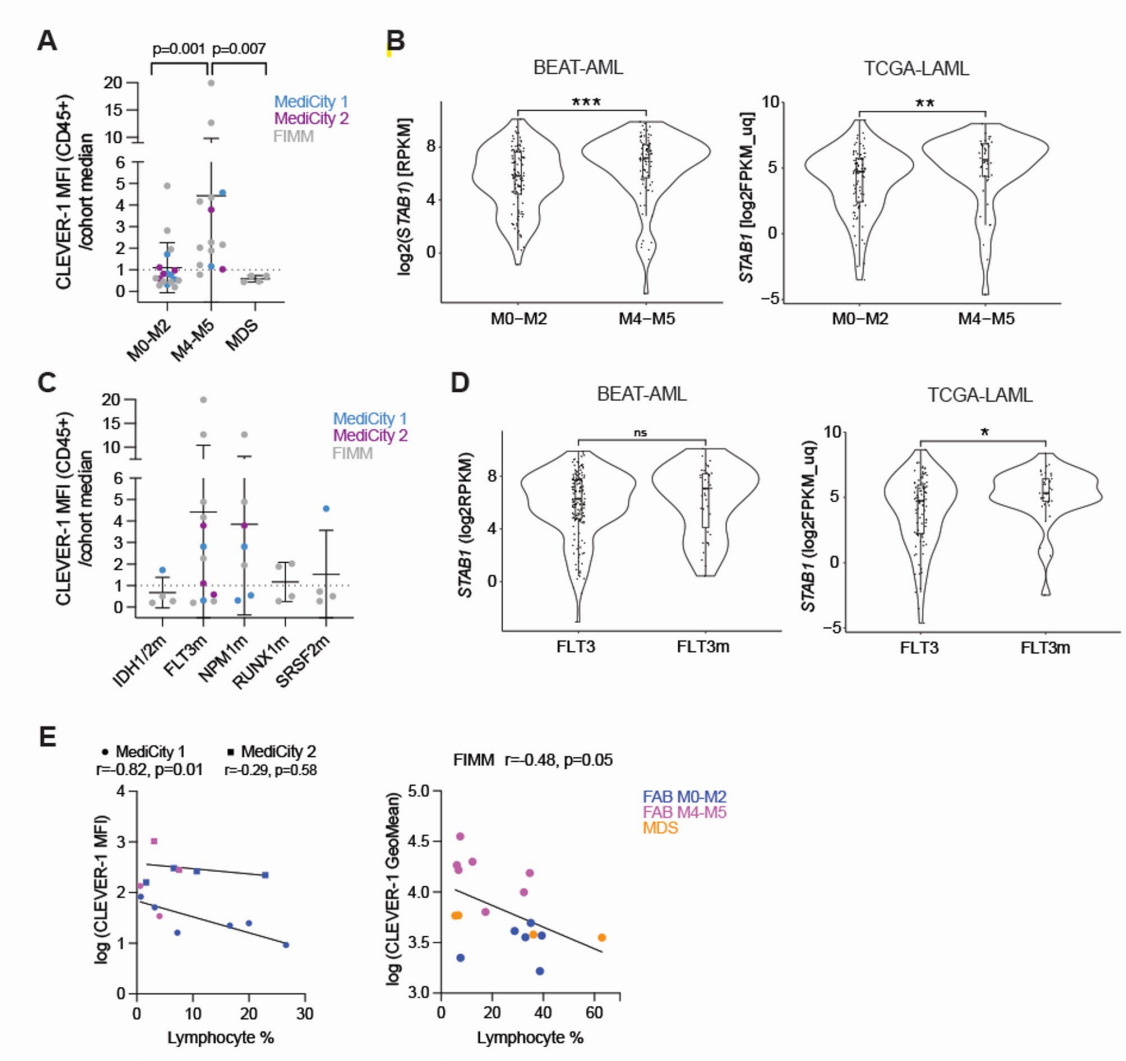
High CLEVER-1 levels in AML with monocytic differentiation. (**A**) Total CLEVER-1 expression (in CD45 + cell population) in each analyzed sample, normalized to sample cohort median, grouped by disease and AML FAB type. (**B**) Violin plots showing *STAB1* RNA expression in BEAT-AML (*n* = 192) and TCGA-AML (*n* = 132) patient datasets categorized by FAB class. or (**C**) CLEVER-1 expression grouped by myeloid mutations present in the analyzed samples. (**D**) Violin plots showing *STAB1* RNA expression in BEAT-AML and TCGA-AML datasets according to FLT3 mutation status. In A and C, each dot represents a sample, line shows mean +/- s.d. Sample cohorts are indicated by different colors. In C, one sample can appear in multiple groups. Only mutations present in at least 10% of the samples, were included. P values are based on Kruskal-Wallis and Dunn’s test. For B and D, box plots display median and interquartile range. Wilcoxon rank-sum test. (**E**) Pearson correlation of lymphocyte frequency (% from CD45 + cells) and total CLEVER-1 expression in MediCity (MediCity 1 = circle, MediCity 2 = square) and FIMM cohorts. Each sample is a dot and color indicates FAB type/disease. ***, *P*-value < 0.001; **, *P*-value < 0.01; * *P*-value < 0.05; NS, not significant.

In accordance with the high CLEVER-1 expression in monocyte cell populatios, comparison of total CLEVER-1 level between different AML FAB types and MDS showed significantly higher total CLEVER-1 level in myelomonocytic/monocytic AML (FAB M4-M5) compared to FAB M0-M2 AML or MDS, among primary patient samples from all three sample cohorts (Fig. 2A and Fig. S2C). Analysis of *STAB1* mRNA expression from the BEAT-AML 2.0^19^ and TCGA-LAML^20^ datasets confirmed significantly higher *STAB1* mRNA levels in M4/M5 AML compared to the M0-M2 group (Fig. 2B)^19^. In addition, we observed a trend for higher *STAB1* mRNA expression in Fms Related Receptor Tyrosine Kinase 3 (FLT3) and/or Nucleophosmin 1 (NPM1) mutated AML compared to the other most frequent mutations (Isocitrate Dehyrogenase 1/2 (IDH1/2), RUNX1, Serine/arginine rich splicing factor 2 (SRSF2)) in the tested samples (Fig. 2C). Higher *STAB1* mRNA expression in FLT3-ITD mutated AML was confirmed in TCGA-LAML data but showed no statistical significance in the BEAT-AML dataset (Fig. 2D)^13^. In addition, CLEVER-1 protein expression had a significant negative correlation with the percentage of lymphocytes, independent of the sample’s blast percentage, in the two sample cohorts (MediCity 1, FIMM) that measured CLEVER-1 expression from the cell surface (Fig. 2E). Altogether, these results validate CLEVER-1 expression beyond tumor-associated macrophages in the context of myeloid malignancies, especially in AML with monocytic differentiation or low bone marrow lymphocyte percentage.

### HLA-DR expression is increased in AML and MDS bone marrow samples treated ex vivo with anti-CLEVER-1 antibody, bexmarilimab

**Fig. 3:**
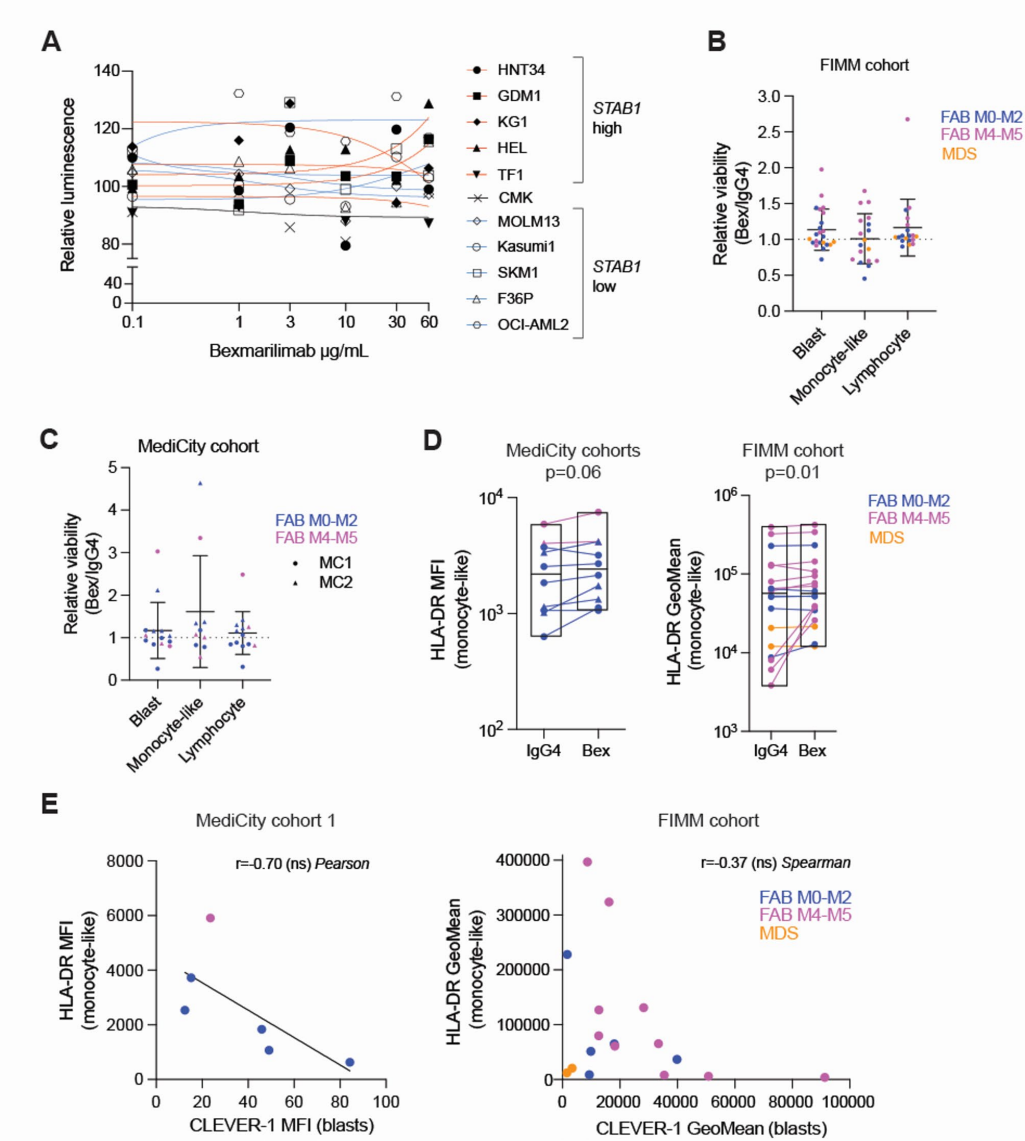
HLA-DR expression induction in monocyte-like cells upon ex vivo bexmarilimab treatment. (**A**) Relative luminescence (versus IgG4 treated), indicating the amount of ATP in AML cell lines after 72 h treatment with increasing concentrations of bexmarilimab. Data is from one experiment, mean calculated from technical replicates (*n* = 2/concentration). Red lines = *STAB1*^high^ cell lines, black line = *STAB1*^mid^ cell line, blue lines = *STAB1*^low^ cell lines. (**B**) Relative viability (normalized to IgG4) of primary bone marrow cell populations after 48 h bexmarilimab (Bex) treatment from FIMM cohort samples and from (**C**) Medicity cohorts’ samples (cohort 1/MC1 = circle, cohort 2/MC2 = triangle). Samples are labeled with colour according to FAB subtype/disease, mean and sd per cell population is indicated. Dashed line represents IgG4 control. Only cell populations with enough cells were included in the analysis (≥ 5% of parent population or > 100 events in IgG4-treated at 48 h). (**D**) HLA-DR expression (Median Fluorescence Intensity, MFI or Geometric Mean Intensity, GeoMean) on monocyte-like cells after 48 h treatment with 50 µg/mL IgG4 (irrelevant human IgG4, isotype control) or bexmarilimab (Bex) in all three sample cohorts. P-values derived from paired t-test (MediCity cohorts) or Wilcoxon signed rank test (FIMM cohort). The results from one sample are connected by a line and boxplots show treatment group mean (min, max). Only samples with enough monocyte-like cells were included in the analysis (≥ 5% of parent population or > 100 events in IgG4-treated at 48 h). Samples are labeled with colour according to FAB subtype/disease and MediCity cohort 1 and 2 samples marked by different shapes. (**E**) Correlation plots of blast cell surface CLEVER-1 expression versus HLA-DR expression in monocyte-like cells from MediCity cohort 1 and FIMM cohort. Samples are labeled with colour according to FAB subtype/disease.

Treatment of AML cell lines with up to 60 µg/mL of the CLEVER-1 blocking therapeutic antibody, bexmarilimab, did not decrease cell viability (Fig. 3A). Since cell lines lack other immune cells that are present in the leukemic bone marrow and may contribute to bexmarilimab response, we treated the 34 AML and 4 MDS bone marrow samples ex vivo with bexmarilimab as a single-agent for 48 h and used flow cytometry profiling to study the effect of CLEVER-1 inhibition in primary leukemia and MDS cells.

In the primary AML and MDS samples, no significant effect on cell viability was observed by bexmarilimab (50 µg/mL) compared to IgG4 but individual samples showed alterations in viability after bexmarilimab treatment in both FIMM and MediCity cohorts (Fig. 3B-C). A notable effect in ex vivo bexmarilimab treated patient samples was a significant increase of the antigen presenting MHC II protein, human leukocyte antigen DR isotype (HLA-DR), expression by monocyte-like cells in one-third to two-thirds of samples with evaluable monocyte population, across cohorts (Fig. 3D). The average increase of HLA-DR per sample cohort varied from 1.2- to 2-fold. Few samples in all cohorts showed concomitant HLA-DR increase in blasts (Fig. S3A). Interestingly, three FIMM cohort samples with low basal HLA-DR expression, showed a dramatic (5-10x) upregulation of HLA-DR. All three samples (FIMM IDs: 8, 14, 9) with the outstanding HLA-DR response were from newly diagnosed, myelomonocytic/monocytic AML and two of these samples had extremely high CLEVER-1 levels (FIMM IDs: 14, 9). Blast CLEVER-1 protein expression showed a negative, yet non-significant, correlation with HLA-DR, in antigen-presenting cells of the bone marrow (Fig. 3E). Bexmarilimab-mediated IFNγ induction is a potential mechanism for the observed HLA-DR increase^9,12,21–24^. From the 14 primay AML samples tested at MediCity, 9 samples had enough material to be tested for IFNγ induction by ex vivo bexmarilimab treatment. 5/9 samples showed increased IFNγ levels after bexmarilimab treatment compared to IgG4 treated control (Fig. S4A). Only two of these five samples had HLA-DR expression data available, with one sample showing increased monocyte-like cell HLA-DR levels after ex vivo bexmarilimab treatment. We sought more evidence on bexmarilimab-mediated induction of IFNγ in AML by using the AML cell line THP-1 with a NF-κB luciferase reporter, which is a tool for studying IFNγ induced signalling pathways. Bexmarilimab showed a dose-dependent effect on NF-κB signalling activation, which was reduced to half by addition of a competing recombinant CLEVER-1 fragment (Fig S4B-C). In conclusion, ex vivo bexmarilimab single-agent does not have a cytotoxic effect on AML/MDS blast cells but MHC II expression in antigen-presenting cells is increased upon bexmarilimab treatment, especially in samples with low HLA-DR and high CLEVER-1 expression.

### Bexmarilimab plus azacitidine supports MHC II protein expression in AML and MDS bone marrow cells ex vivo

**Fig. 4:**
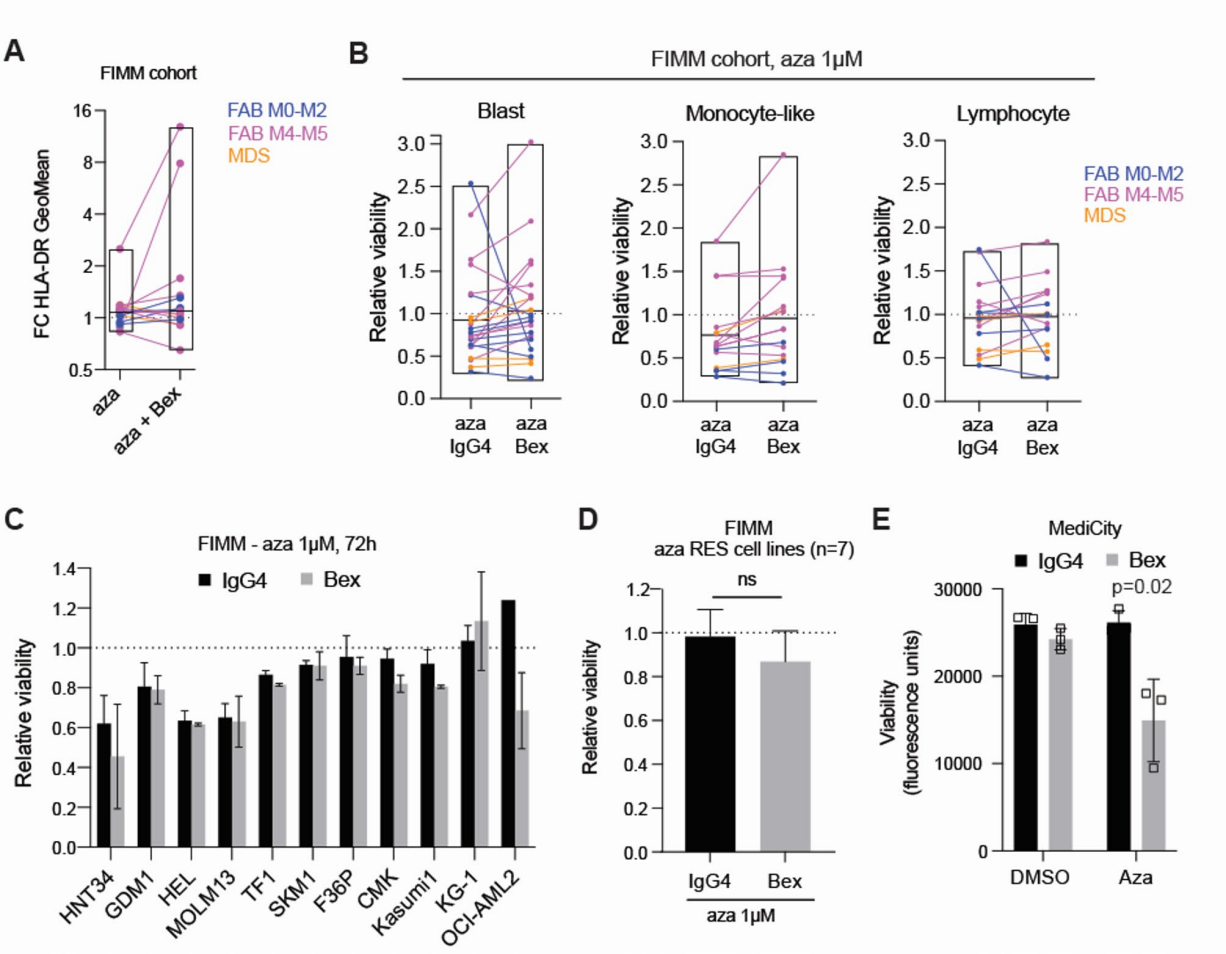
Antigen presenting molecule expression is increased after treatment with bexmarilimab with and without azacitidine. (**A**) Fold change (FC) of HLA-DR expression after treatment with 300nM azacitidine in combination with IgG4 or bexmarilimab, both compared to IgG4 treatment, in FIMM cohort samples. The results from one sample are connected by a line and boxplots show treatment group mean (min, max). Samples with sufficient total viable cell yield after thawing were tested with the combination treatment and only samples with enough monocyte-like cells were included in the analysis (≥ 5% of parent population or > 100 events in IgG4-treated at 48 h). Bex = bexmarilimab (50 µg/mL), aza = azacitidine (300 nM). Samples are labeled with colour according to FAB subtype. (**B**) Relative viability (normalized to IgG4) of primary bone marrow cell populations after 48 h azacitidine (aza; 1 µM) plus IgG4 or bexmarilimab (Bex) treatment, in FIMM cohort samples Samples are labeled with colour according to FAB subtype/disease, results from one sample are connected by a line and boxplots show mean (min/max) per treatment group. Dashed line represents IgG4 control. Only cell populations with enough cells were included in the analysis (≥ 5% of parent population or > 100 events in IgG4-treated at 48 h). (C**C**) Relative viability (normalized to IgG4), measured by Cell Titer Glo at FIMM, after 72 h 1 μm azacitidine plus IgG4 or bexmarilimab (Bex) treatment of 11 AML cell lines and (**D**) pooled of 7 AML cell lines that were resistant to azacitidine (TF1, SKM1, F36P, CMK, Kasumi1, KG-1, OCI-AML2; >80% relative viability). For C) all cell lines were tested once, average and standard deviations are calculated from technical replicates (*n* = 2). Statistical significance for D was tested with paired t-test (*n* = 7). (**E**) Fluoresence, indicating cell viability, measured at MediCity with Alamar Blue, of KG1 AML cell lines, after 7 day treatment with bexmarilimab or IgG4 plus 1 μm azacitidine or DMSO. Data is presented as mean +/- sd from three replicate experiments.

The hypomethylating agent azaciditine, used as a SoC for MDS and AML, is known to have immunomodulatory effects, such as increased expression of antigen presenting molecules and tumor antigens^21,24–26^, which could be favorable when combined with immune activating therapeutics. Our e*x vivo*, single-agent, short (48 h) azacitidine (300 nM) treatment of the AML/MDS bone marrow samples (FIMM cohort) increased HLA-DR expression only slightly in monocyte-like cells (Fig. 4A; avg 1.15-fold compared to IgG4). Interestingly, in 5/15 (33%) patient samples, the azacitidine-induced HLA-DR increase was enhanced by azacitidine + bexmarilimab, in average by 3.6-fold (range 1.1-9.0-fold), compared to azacitidine alone, although the comparison of treatment group means did not show a significant difference (Fig. 4A). The five samples that were sensitive for further HLA-DR increase by bexmarilimab + azacitidine included all samples with NRAS mutation (*n* = 3) within the tested sample set. Additionally, 4/5 samples had either mutated NPM1 or FLT3, or FAB M4-M5 subtype, which were features of AML samples with high *STAB1* mRNA expression (Fig. 2B-C).

Treatment of primary AML and MDS samples with higher concentration (1 µM) azacitidine (FIMM cohort) for 48 h reduced blast cell viability in 12/22 evaluable samples by at least 20% (avg viability 0.59 +/- 0.15) (Fig. 4B). In 2 samples (FIMM IDs: 4, 10), blast cell viability was reduced slightly further by bexmarilimab + azacitidine combination. In addition, blast cell viability was reduced more by the combination in 3 samples (FIMM IDs: 13, 15, 7) where azacitidine alone seemingly increased blast cell viability (Fig. 4B). Treatment with a lower concentration of azacitidine (300 nM) was overall less cytotoxic, with reduced blast viability in 6/22 samples by at least 20% (avg viability 0.57 +/-0.16), but combination of bexmarilimab was as effective with both tested azacitidine concentrations based on further reduction of cell viability in 2/6 samples sensitive to 300nM azacitidine and in 3 samples not responding to 300nM azacitidine (Fig. S3B). Compared to the blast cells, the monocyte-like population was more sensitive to 1 µM azacitidine, with 12/16 evaluable samples showing at least 20% reduction in cell viability (avg viability 0.56 +/- 0.17) (Fig. 4B). Bexmarilimab + azacitidine combination rather increased than decreased cell viability in monocyte-like cells, with 5/12 azacitidine sensitive samples not showing decrased monocyte viability upon bexmarilimab + azacitidine treatment (Fig. 4B). Lymphocyte viability was decreased by at least 20% only in 5/17 evaluable samples by azacitidine (avg viability 0.56+/- 0.14). Bexmarilimab + azacitidine reduced lymphocyte viability in 2 samples (FIMM IDs: 13, 15), in which lymphocytes were resistant to azacitidine alone (Fig. 4B).

In the AML cell lines tested at FIMM, a high concentration of azacitidine (1 µM) had a cytotoxic effect after 72 h in 4/11 cell lines (HNT34, GDM1, HEL, MOLM13; avg viability 0.68 +/0.09) (Fig. 4C). Only the OCI-AML2 cell line showed a tendency for sensitization to azacitidine by bexmarilimab (Fig. 4C). Pooling of the 7 cell lines that showed > 80% cell viability after IgG4 + azacitidine treatment, did not show significant enhancement of cytotoxicity by bexmarilimab + azacitidine (Fig. 4D). At MediCity, in a 7-day culture using StemSpan medium and the KG-1 cell line, a significant reduction of cell viability in the bexmarilimab + azacitidine combination was seen, compared to the modest cytotoxic effect of 1 µM azacitidine alone (Fig. 4E). In conclusion, bexmarilimab plus azacitidine enhance antigen presentating molecule expression in a subset of primary AML/MDS cells. The drug combination’s effect on malignant blast cell viability varied between individuals when tested with primary samples and depending on cell line culture conditions, with enhanced reduction of cell line viability by the combination compared to azacitidine alone in longer culture.

### Bexmarilimab in combination with azacitidine/venetoclax suppresses cell viability in venetoclax-resistant cell lines

**Fig. 5:**
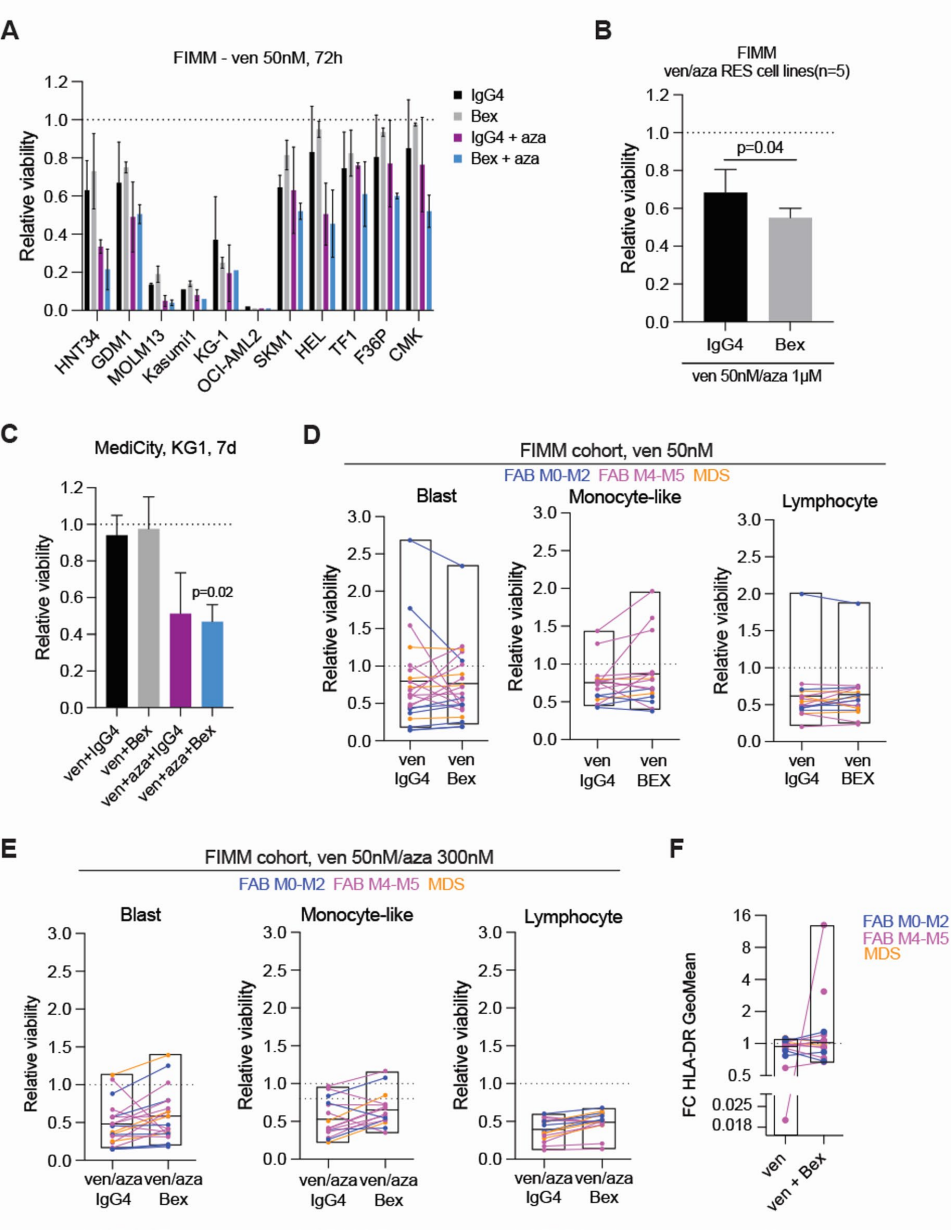
Bexmarilimab overcomes venetoclax resistance in AML cell lines. (**A**) Relative viability (normalized to IgG4), measured by Cell Titer Glo at FIMM, of 11 AML cell lines after 72 h treatment with 50nM venetoclax (ven) or venetoclax/azacitidine (aza; 1 µM) plus IgG4 or bexmarilimab (Bex) and (**B**) pooled of 5 AML cell lines that were resistant to venetoclax/azacitidine (GDM1, SKM1, TF1, F36P, CMK; >50% relative viability). For A) all cell lines were tested once, average and standard deviations are calculated from technical replicates (*n* = 2). Statistical significance for B was tested with paired t-test (*n* = 5). (**C**) Relative viability (normalized to IgG4), measured at MediCity with Alamar Blue, of KG1 AML cell lines, after 7 day treatment with venetoclax (50 nM) or venetoclax/azacitidine (1 µM) plus bexmarilimab (Bex) or IgG4. Data is presented as mean +/- sd from three replicate experiments. P value indicates difference between ven + aza + Bex and IgG4. (** D**) Relative viability (normalized to IgG4) of primary bone marrow cell populations after 48 h venetoclax (ven; 50 nM) or (**E**) venetoclax/azacitidine (300 nM) plus IgG4 or bexmarilimab (Bex) treatment, in FIMM cohort samples. Samples are labeled with colour according to FAB subtype/disease, results from one sample are connected by a line and boxplots show treatment group mean (min, max). Dashed line represents IgG4 control. Only cell populations with enough cells were included in the analysis (≥ 5% of parent population or > 100 events in IgG4-treated at 48 h). (**F**) Fold change (FC) of HLA-DR expression in FIMM cohort samples after treatment with venetoclax (5 nM) plus IgG4 or bexmarilimab, both compared to IgG4 treatment. The results from one sample are connected by a line and boxplots show treatment group mean (min, max). Only samples with enough monocyte-like cells were included in the analysis (≥ 5% of parent population or > 100 events in IgG4-treated at 48 h).

The Bcl-2 inhibitor venetoclax has shown promising clinical efficacy in AML, especially in the previously untreated patients in combination with azacitidine^27^ and knockdown of *STAB1* has been suggested to sensitize AML cell lines to venetoclax^13^. Thus, we tested bexmarilimab in combination with venetoclax and venetoclax/azacitidine in AML cell lines and AML/MDS bone marrow samples (FIMM cohort). Nearly all cell lines tested at FIMM responded to venetoclax (50 nM) and/or venetoclax (50nM)/azacitidine (300 nM). Bexmarilimab did not improve the effect of venetoclax but reduced cell viability as a triplet with venetoclax/azacitidine (Fig. 5A), with statistical significance in pooled analysis of the 5 cell lines (GDM1, SKM1, TF1, F36P, CMK) that showed > 50% viability remaining after venetoclax/azacitidine/IgG4 treatment (Fig. 5B). At MediCity, treatment of the KG-1 cell line with venetoclax and venetoclax/azacitidine +/- bexmarilimab for 7 days did not show a significant difference in viability between IgG4 and bexmarilimab combinations but the venetoclax/azacitidine/bexmarilimab triplet reduced cell viability compared to IgG4 alone (Fig. 5C). Notably, the StemSpan medium used in this culture setting may not be optimal for studying venetoclax sensitivity^16^. From the FIMM cohort patient samples, in 5/18 AML (FIMM IDs: 13, 7, 19, 15, 16) and 2/4 (FIMM IDs: 20, 25) MDS samples the blast population was ex vivo resistant to 50 nM venetoclax (> 0.80 relative viability) and in three samples also to venetoclax (50nM)/azacitidine (300 nM) (FIMM IDs: 7, 19, 20) (Fig. 5D-E). Addition of bexmarilimab to venetoclax sensitized the blasts in 2/5 venetoclax resistant AML samples (FIMM IDs: 19, 15; avg viability 0.49 +/-0.08) and reduced blast cell viability further in 2/13 venetoclax sensitive AML samples (FIMM IDs: 8, 17) (Fig. 5D). Among the three venetoclax (50nM) + azacitidine (300nM) resistant AML samples, only one (FIMM ID: 19) was sensitized to blast killing by bexmarilimab (Fig. 5E). The resistance of monocyte-like cells to venetoclax was observed mainly in the same samples as blast cell resistance. Bexmarilimab did not sensitize the resistant monocyte-like cells to venetoclax, but the combination reduced monocyte-like cell viability further in 4 AML samples (FIMM IDs: 10_1, 14, 17, 8) where venetoclax alone was already effective (Fig. 5D). Addition of bexmarilimab to venetoclax (50nM)/azacitidine (300nM) rather increased the viability of monocyte-like cells (Fig. 5E). Only in 1/17 evaluable samples (FIMM ID: 13), which showed high relative viability upon all treatments, lymphocytes were ex vivo venetoclax resistant (Fig. 5D). Bexmarilimab reduced lymphocyte viability slightly further in 1 MDS (FIMM ID: 27) and 2 AML (FIMM IDs: 15, 17) samples (Fig. 5D) but rather increased lymphocyte viability as a triplet with venetoclax (50nM)/azacitidine (300nM) (Fig. 5E). However, comparison of the treatment groups did not show significant difference for any of the cell populations.

Finally, to study venetoclax/bexmarilimab effect on HLA-DR expression, low concentration (5 nM) venetoclax was used. Venetoclax decreased HLA-DR levels in 3/15 (FIMM IDs: 14, 2, 17) samples but bexmarilimab + venetoclax combination treatment still resulted in upregulation of HLA-DR in 4/15 samples (FIMM IDs: 14, 9, 10_1, 19), of which one (FIMM ID: 14) had the most significant decrease of HLA-DR by venetoclax alone (Fig. 5F). Collectively, combining bexmarilimab with venetoclax does not improve bexmarilimab’s HLA-DR upregulating effect but sensitized 33–40% of ex vivo resistant AML samples to venetoclax or venetoclax/azacitidine without inducing further lymphocyte toxicity. Moreover, bexmarilimab/azacitidine/venetoclax triplet reduced the viability of AML cell lines.

## Discussion

Taken together, we show that the macrophage checkpoint protein CLEVER-1 is present on the surface of immature myeloid cells (blasts, CD34 + progenitors) and monocytes derived from the immature malignant cells, in the bone marrow of AML and MDS patients. Due to the abundance of CLEVER-1 on monocytes, primary AML samples with monocytic differentiation showed highest CLEVER-1 levels. The lack of correlation between *STAB1* mRNA or CLEVER-1 protein expression and FAB type in AML cell lines suggests that the blast/monoblast cell CLEVER-1 expression is independent of FAB type. Interestingly, high CLEVER-1 may be associated with FLT3 mutation, as previously reported for *STAB1* mRNA in cytogenetically normal AML^13^. The observed trend of high leukemic cell CLEVER-1 association with low HLA-DR expression and lymphocyte abundance in the BM, is in line with the immunosuppressive role of tumor-associated macrophage CLEVER-1 in solid tumors^7,9,12^ but requires further studies to validate the functional relevance of this association in myeloid malignancies.

Despite the potential of M2-like macrophages to support leukemia cell growth^3–6^, here we focused on studying the effects of bexmarilimab on malignant myeloid cells since the anti-tumor effects of inhibiting macrophage CLEVER-1 have been extensively characterized^7–9,12^. MHC II expression may enable improved recognition of leukemic cells by the immune system and decrease the likelihood of disease relapse after allogeneic stem cell transplant^23^ and is a known effect of azacitidine as well as seen in CLEVER-1 deficient mice^10,21,24–26^. Induction of IFNγ signaling by both bexmarilimab and azacitidine^9,21,24^, known to induce MHC expression^22,23^, is a potential mechanism. Indeed, MHC II expression on antigen presenting cells was increased in 39% of all MDS and AML samples after inhibition of CLEVER-1 with ex vivo bexmarilimab treatment. Furthermore, in one-third of the the FIMM cohort primary samples, HLA-DR expression was further increased after bexmarilimab plus azacitidine treatment compared to azacitidine alone. Induction of IFNγ signaling by both drugs^9,21^, known to induce MHC expression^26^, may explain the combined effect. In combination with the Bcl-2 inhibitor venetoclax, bexmarilimab increased HLA-DR in 27% of the FIMM cohort samples, although HLA-DR levels were downregulated in 20% of the samples by venetoclax, as previously described in venetoclax-treated mouse bone marrow^28^. The induction of MHC II expression by bexmarilimab and bexmarilimab plus azacitidine may promote antigen presentation. However, no functional validation was performed in this study but enhanced antigen cross-presentation has been previously shown in bexmarilimab treated macrophages^9^.

Bexmarilimab does not bind Fc receptor and, thus is unlikely to trigger antibody-dependent cellular cytotoxicity^8^. As expected, bexmarilimab as a single-agent did not have immediate (48–72 h treatment) cytotoxic effect in any of the tested AML cell lines and reduced blast cell viability slightly in only 5% of all ex vivo tested AML/MDS patient samples. Previously reported enhanced sensitivity of AML cell lines to venetoclax after silencing of CLEVER-1 suggests that CLEVER-1 inhibition may increase the sensitivity of malignant blasts to cytotoxic agents^13^. Addition of bexmarilimab to azacitidine or venetoclax reduced ex vivo blast cell viability more than the SoC agents alone, in 23% and 27% of the FIMM AML/MDS samples, respectively. These samples were variable in terms of FAB subtype and CLEVER-1 protein expression but the majority of these samples had myeloid mutations related to transcription, translation, DNA methylation and chromatin. In AML cell lines, bexmarilimab plus azacitidine showed cytotoxicity only after a longer, 7-day culture. However, the triplet combination of bexmarilimab with azacitidine and venetoclax reduced the viability of venetoclax/azacitidine resistant AML cell lines in a 3-day culture as well as KG-1 cell line viability after a 7-day culture. Despite the variability, these results suggest that bexmarilimab has potential to increase the susceptibility of malignant blasts to venetoclax and/or azacitidine induced cell death. A potential mechanism may be modulation of the metabolic fitness of the blasts as respiratory electron transport chain genes were found to be downregulated in tumor-associated macrophages of patients with ER + breast cancer or biliary tract cancer who responded to bexmarilimab treatment^12^ and AML blast’s sensitivity to venetoclax is known to depend on e.g. the metabolic state of the cells^29,30^.

Finally, whereas the effect of bexmarilimab on the proliferation of monocyte-like cells in the primary samples was variable, lymphocyte cell viabibility was increased in 28% of the primary samples upon bexmarilimab treatment. Support of the bone marrow lymphocyte population, even in combination with lymphotoxic venetoclax, may be an additional anti-leukemic effect of bexmarilimab, enhancing malignant cell targeting by the bone marrow immune system and supporting bone marrow recovery.

The effects of bexmarilimab alone and in combination with SoC in this study were mainly observed in a subset of the primary samples and potential association of e.g. HLA-DR increase with clinical sample feature(s) was challenging to assess from this limited set of samples. In general, the four MDS samples included in this study showed milder changes in response to bexmarilimab than AML patient samples. However, this difference would need to be validated with a larger set of MDS samples and the possibility that the blast cells derived from AML and MDS patient’s may behave differently in the ex vivo culture setting needs to be acknowledged as a possible contributing factor.

In conclusion, the results presented here suggest that bexmarilimab can target CLEVER-1 on malignant myeloid cells to potentially increase antigen-presentation capability and promote sensitivity to cytotoxic agents, supporting the ongoing clinical development of bexmarilimab in combination with SoC in myeloid malignancies (NCT05428969).

## Electronic supplementary material

Below is the link to the electronic supplementary material.

Supplementary Material 1

Supplementary Material 2

## Data Availability

The datasets analysed during the current study are available in the HEMAP: Online resource for interactive exploration of hematopoietic cancer data (http://hemap.uta.fi/), BEATAML2, (https://biodev.github.io/BeatAML2/) and Genomic Data Commons (GDC) Data Portal (https://portal.gdc.cancer.gov/projects/TCGA-LAML, https://gdc.cancer.gov/about-data/publications/laml_2012). Other data generated during the current study are available from the corresponding author on reasonable request.
