# Supplementary material for: CLEVER-1 targeting antibody, bexmarilimab, supports HLA-DR expression and alters ex vivo responsiveness to azacitidine and venetoclax in myeloid malignancies
Source: Sci Rep. 2025 May 14;15:16775. doi: 10.1038/s41598-025-01675-y (PMC12078569; doi:10.1038/s41598-025-01675-y)
Supplement: Supplementary file 2 — Supplementary Material 2 [file 41598_2025_1675_MOESM2_ESM.pdf]

Supplementary information

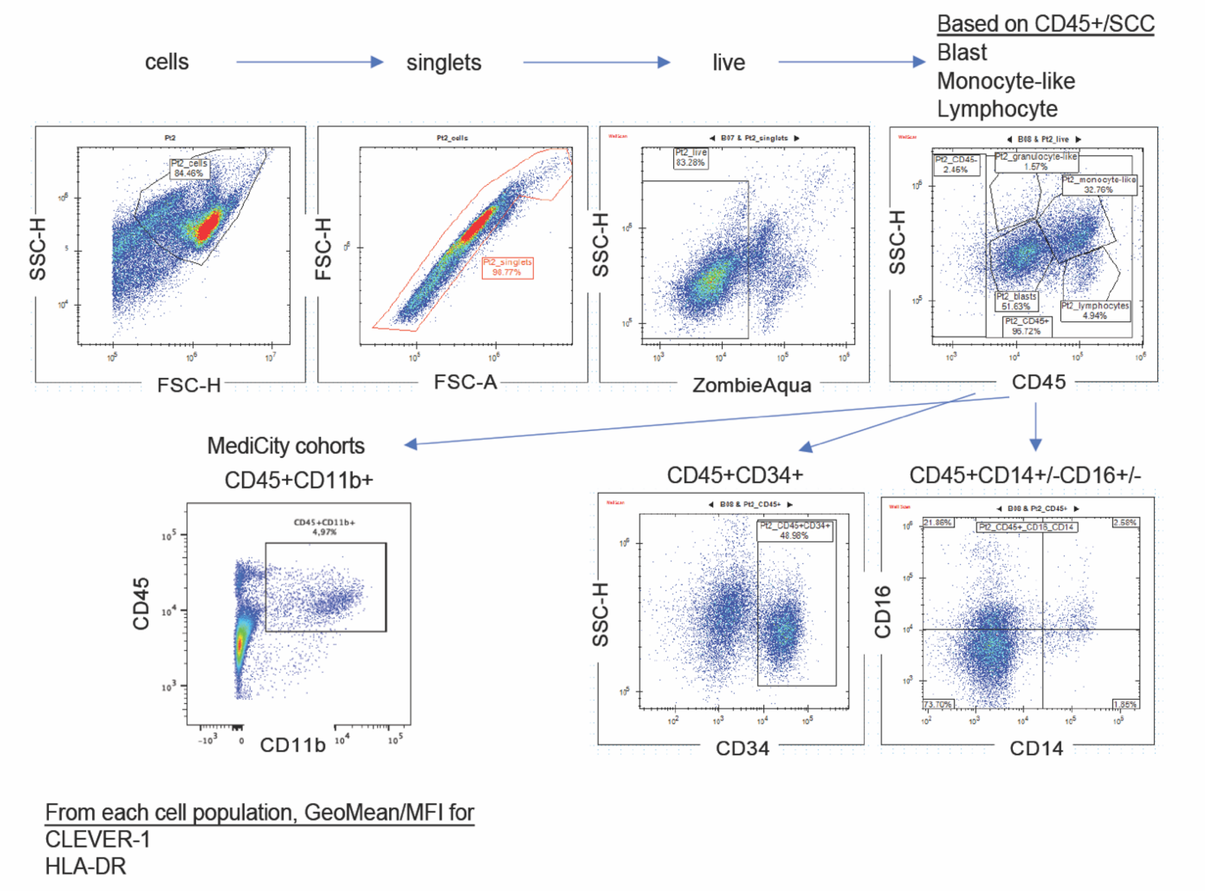

Supplementary Figure S1. Flow cytometry gating strategies.

Schematic representation of the gating strategy of mononuclear cell populations used for primary AML and MDS sample analyses. Clever-1 and HLA-DR expression as mean or median fluorescence intensity from each population was analyzed.

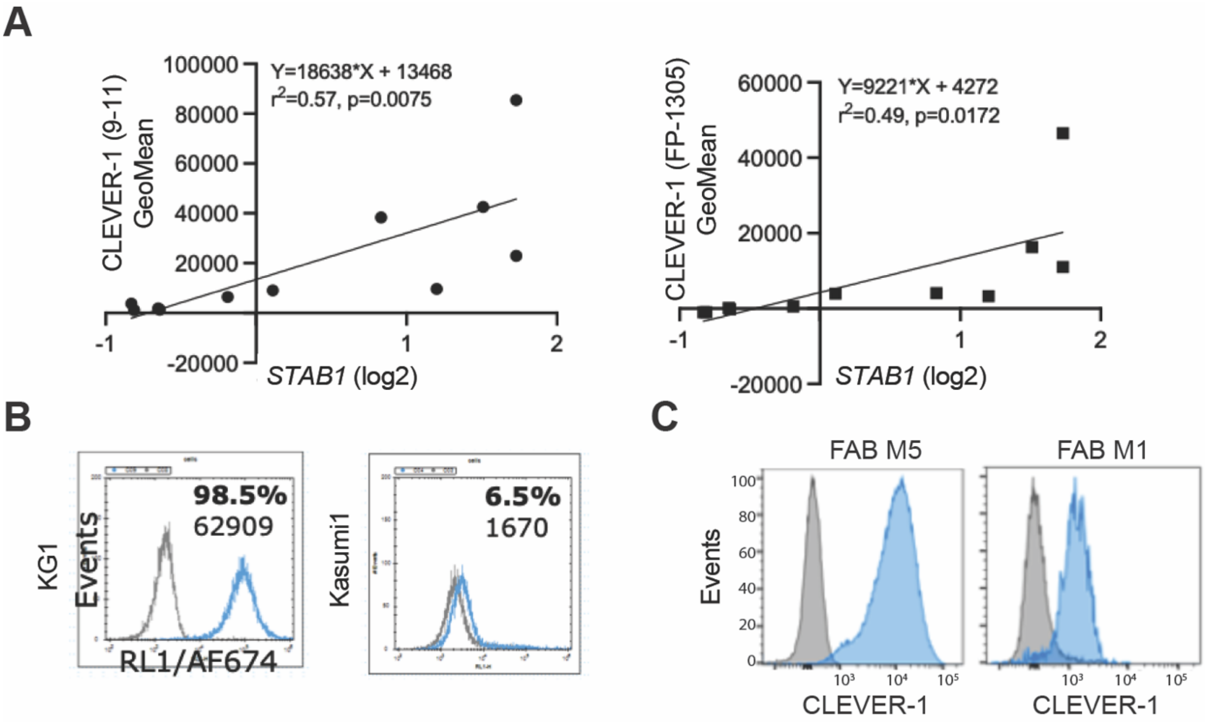

Supplementary Figure S2. Clever-1 expression in AML.

A) Simple linear regression analysis of *STAB1* RNA (log2) and Clever-1 protein (GeoMean) expression in the studied AML cell lines. **B)** Representative flow cytometry histograms of AML cell lines with high (KG-1) and low (Kasumi1) Clever-1 protein expression. Numbers indicate IgG control normalized % of Clever-1+ cells/live cells and GeoMean of Clever-1. **C)** Flow cytometry histograms showing higher Clever-1 protein expression in monocytic AML (FAB M5) compared to AML with minimal maturation (FAB M1) in two representative patient samples from Medicity cohort 2. Grey=IgG control, Blue=Clever-1 antibody

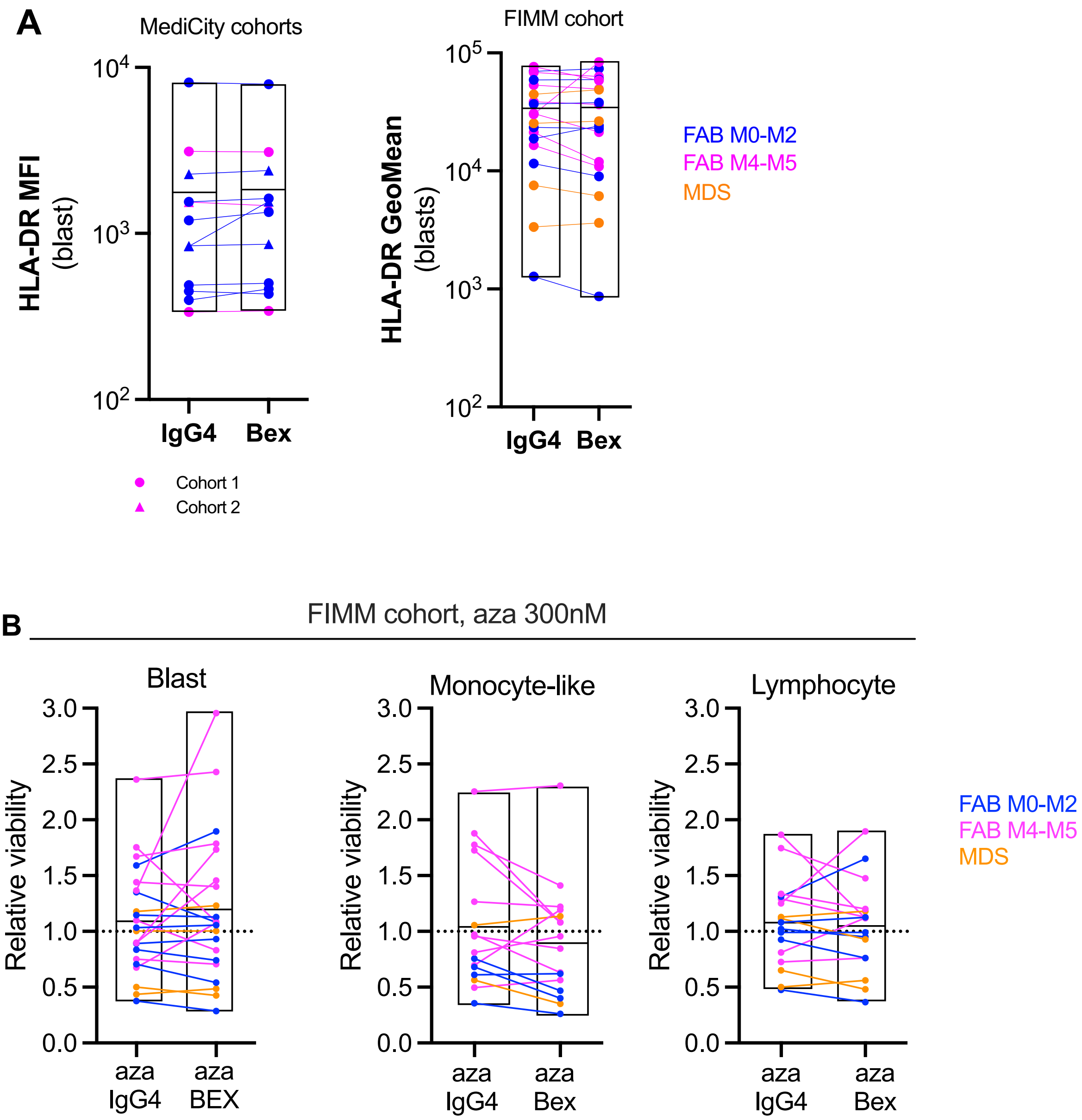

Supplementary Figure S3. Bexmarilimab effects on AML cells.

A) Expression of HLA-DR in blasts after 48h IgG4 or bexmarilimab treatment in all three cohorts (MediCity 1 and 2, FIMM). The results from one sample are connected by a line and boxplots show treatment group mean (min, max). Bex=bexmarilimab (50 ug/mL). Samples are labeled with color according to FAB subtype and MediCity cohort 1 and 2 samples marked by different shapes. **B)** Relative viability (normalized to IgG4) of primary bone marrow cell

populations after 48h azacitidine (aza; 300nM) plus IgG4 or bexmarilimab (Bex) treatment, in FIMM cohort samples Samples are labeled with colour according to FAB subtype/disease, results from one sample are connected by a line and boxplots show mean (min/max) per treatment group. Dashed line represents IgG4 control. Only cell populations with enough cells were included in the analysis (5% of parent population or >100 events in IgG4-treated at 48h).

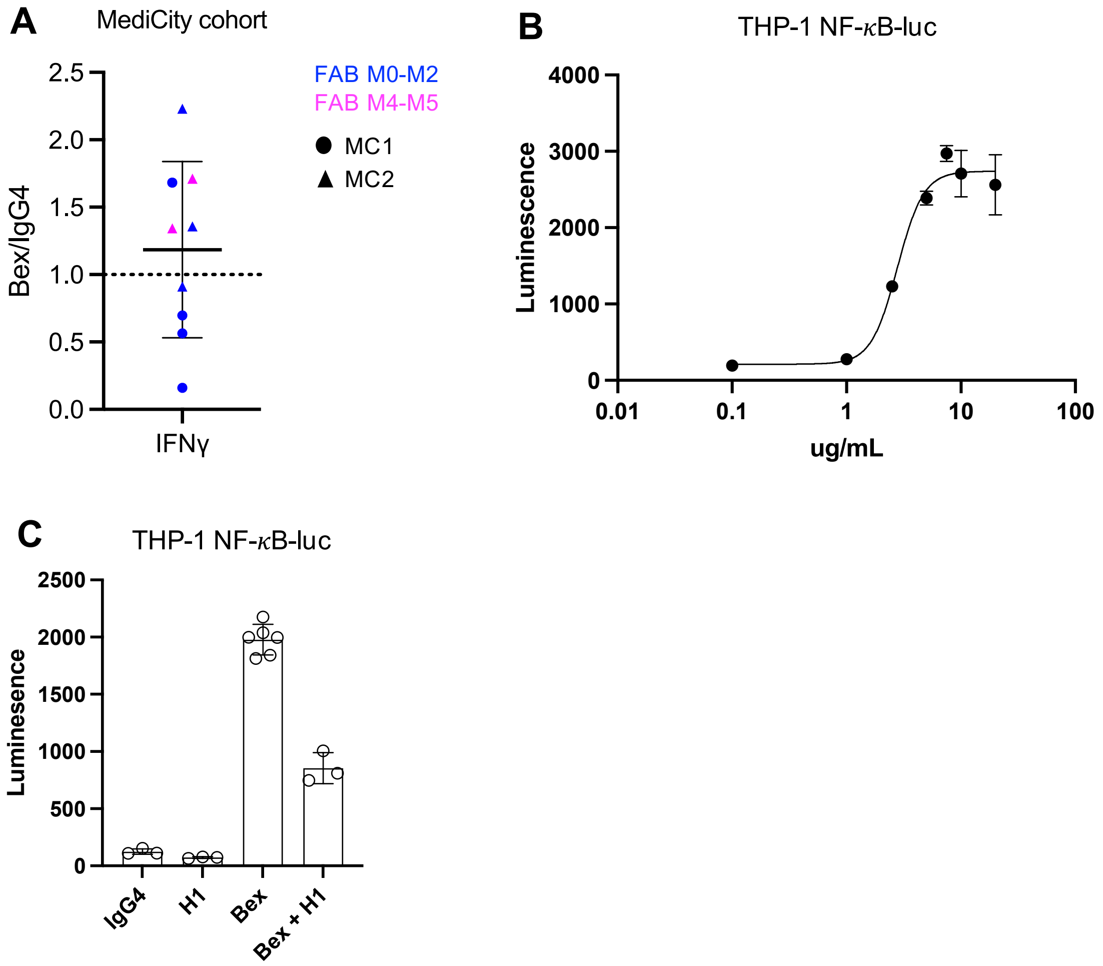

Supplementary Figure 4. Bexmarilimab effect on IFNγ and NF-κB signaling in AML cells. A) Relative interferon gamma (IFNγ) level in the culture medium of primary AML cells from the MediCity sample cohorts after 48h incubation with bexmarilimab (Bex), measured with Multiplex. Samples are labeled with color according to FAB subtype/disease, mean and sd are indicated. Medicity cohorts' samples (cohort 1/MC1=circle, cohort 2/MC2=triangle). Dashed line represents IgG4 control. **B)** Luminescence signal measured from the THP1-Lucia™ NF-κB reporter cell line after bexmarilimab treatment (0.1-20ug/mL). Using sigmoidal 4PL non-linear fit an EC50 value of 2.78 µg/mL was obtained. **C)** Luminescence signal after pre-incubation with a recombinant fragment of Clever-1 (H1; 20 ug/mL) and 10 ug/mL bexmarilimab (Bex) or IgG4 control. Error bars in panel B and data points in panel C represent technical replicates (n=3 in panel B).

Supplementary Table 1. Patient sample characteristics.

Provided as a separate .xls file

Supplementary Table 2. Flow cytometry antibody panels.

Antigen-fluorochrome	Manufacturer	Cat#	Used at	Sample cohort
CD45-PerCp-Cy5.5	BD BioSciences	564105	1:100	MediCity 1 and 2
CD11b-BV650	BD BioSciences	742640	1:100	MediCity 1 and 2
CD14-Pacific Blue	BD BioSciences	558121	1:100	MediCity 1 and 2
CD16-BV786	BD BioSciences	563690	1:100	MediCity 1 and 2
CD34-PE	Biolegend	343606	1:100	MediCity 1 and 2
HLA-DR FITC	BD BioSciences	555558	1:100	MediCity 1
HLA-DR PE-Cy7	BD BioSciences	560651	1:100	MediCity 2
CD45-Bv786	BD BioSciences	563716	1ug/ml	FIMM
CD34-PE	BD BioSciences	560941	0.13ug/ml	FIMM
CD16-Bv605	BD BioSciences	563172	1ug/ml	FIMM
CD14-FITC	BD BioSciences	345784	0.25ug/ml	FIMM
HLA-DR-Bv421	BD BioSciences	562804	0.4ug/ml	FIMM
PD-L1-APC	BioLegend	329708	2ug/ml	FIMM
Clever-1 9-11-AF647	MediCity		10ug/ml/1:100	MediCity 1 and 2, FIMM
Clever-1 FP1305-AF647	MediCity		20ug/ml/1:100	MediCity 1 and 2, FIMM
